# Kidney Expression of Toll Like Receptors in Lupus Nephritis: Quantification and Clinicopathological Correlations

**DOI:** 10.1155/2016/7697592

**Published:** 2016-08-21

**Authors:** Fabrizio Conti, Francesca Romana Spinelli, Simona Truglia, Francesca Miranda, Cristiano Alessandri, Fulvia Ceccarelli, Michele Bombardieri, Konstantinos Giannakakis, Guido Valesini

**Affiliations:** ^1^Department of Internal Medicine and Medical Specialties-Rheumatology, Sapienza University of Rome, 00161 Rome, Italy; ^2^Centre for Experimental Medicine and Rheumatology, William Harvey Research Institute, Queen Mary University of London, London EC1M 6BQ, UK; ^3^Department of Radiology, Oncology and Pathology, Sapienza University of Rome, 00161 Rome, Italy

## Abstract

*Objective*. The study aimed at locating and quantifying Toll Like Receptor (TLR) 3, 7, 8, and 9 expression in kidney of patients with lupus nephritis (LN) and correlating them with clinicopathological features.* Methods*. Kidney sections from 26 LN patients and 4 controls were analyzed by immunohistochemistry using anti-human TLR3, TLR7, TLR8, and TLR9 polyclonal antibodies; the number of TLR-positive nuclei/mm^2^ was evaluated on digitalized images.* Results*. Compared to controls, LN showed a significantly higher amount of glomerular and tubulointerstitial TLR9 (*p* = 0.003 and *p* = 0.007), whole and tubulointerstitial TLR3 (*p* = 0.026 and *p* = 0.031), and a higher tubulointerstitial TLR7 (*p* = 0.022). TLR9 positively correlated with activity index (*p* = 0.0063) and tubular TLR7 with chronicity index (*p* = 0.026). TLR9 positively correlated with Renal-SLEDAI (*p* = 0.01).* Conclusions*. This is the first study quantifying kidney expressions of TLRs in LN patients; the results show an overexpression of TLR3, TLR7, and TLR9 and demonstrate a correlation with clinicopathological indices supporting a role of these mediators in the pathogenesis of LN.

## 1. Introduction

Systemic Lupus Erythematosus (SLE) is a chronic autoimmune disease affecting any organ system. Renal involvement is common in SLE: up to 60% of the patients will develop lupus nephritis (LN) as an initial manifestation of the disease or at some time point during the follow-up; moreover, in a prospective study of 1,000 SLE patients, those who presented with nephropathy at the beginning of the study exhibited significantly lower 10-year survival rates [1]. In addition, about 7–15% of the patients who develop LN will eventually progress to end-stage renal disease [2, 3].

Immune complexes (ICs) containing self-antigens, produced* in situ* or deposited from the bloodstream, activate innate and adaptive arms of immune response. Both humoral and cellular immunity initiate and amplify the inflammatory response within the kidney; innate immunity and resident renal cells further participate in the inflammatory, destructive, and restorative processes. The role of Toll Like Receptors (TLRs) in SLE pathogenesis has raised great interest, particularly of those recognizing nucleic acids, the main antigenic targets in SLE. TLRs are essential modulators of innate immune response by recognizing conserved molecular patterns shared by a variety of microorganisms and other danger signals; TLR3 binds double-stranded RNA, TLR7 and TLR8 bind single-stranded RNA, and TLR9 binds microbial unmethylated cytidine-guanidine repeat sequences (CpG-DNA) [4].

Several studies on murine lupus suggested a role for TLR signaling in LN pathogenesis (reviewed in [4]). However, only few studies evaluated kidney expression of the different TLRs in humans demonstrating an increase of renal TLR3, TLR7, TLR8, and TLR9 in patients with SLE compared with healthy controls and a variable expression in glomeruli and tubules [5–9]. Moreover, a dual TLR7 and TLR9 antagonist demonstrated its efficacy in reducing plasmacytoid dendritic cells (pDC) of SLE patients and lowering IFN-*α*-regulated genes; these data have further been confirmed in murine models [New Zealand Black/New Zealand White (NZB/NZW)] [10].

To the best of our knowledge, previous researches evaluated renal TLRs expressions only semiquantitatively. In the present study, we aimed to locate and to quantify the expressions of TLR3, TLR7, TLR8, and TLR9 in kidney sections of patients with LN and to correlate TLRs expressions with clinical and histological features. Moreover, we looked for possible correlations between TLRs and histological and/or clinical features of LN.

## 2. Patients and Methods

The study was carried out on patients with SLE, diagnosed according to American College of Rheumatology (ACR) revised classification criteria [11] and followed up at Lupus Clinic, Sapienza University of Rome; patients with a clinical suspicion of lupus nephritis requiring a kidney biopsy for standard of care management in order to provide a histological evaluation of the renal involvement were included.

LN was diagnosed and classified according to International Society of Nephrology/Renal Pathology Society 2003 criteria [12]. Activity index and chronicity index were calculated as reported [13].

As control, we used disease-free kidney sections derived from tissue margins of total or subtotal nephrectomies obtained from 4 patients undergoing surgery for renal malignancies.

Patients' demographic, clinical, and serological data, including SLE clinical features, immunological abnormalities, serum creatinine, 24-hour proteinuria, urinary sediment, hypertensive status, dyslipidemia, and concomitant treatment, were recorded in an electronically filled database.

SLE disease activity was calculated using Systemic Lupus Erythematosus Disease Activity Index (SLEDAI) 2000 [14]; renal items of SLEDAI (R-SLEDAI) were considered as a separate score.

All the patients signed a written informed consent. The protocol was approved by Ethical Committee of Sapienza Università di Roma, Azienda Ospedaliera Policlinico Umberto I.

### 2.1. Immunohistochemistry and Light Microscopy

Renal tissue was obtained by percutaneous needle biopsy. Tissue cylinders were received within 15 minutes from the time of biopsy; specimens from each biopsy were divided into three portions. Two were processed for routine light and electron microscopic examination; the third fragment was embedded in Optimal Capture Temperature (OCT) and snap-frozen in liquid nitrogen-cooled isopentane for immunohistochemistry (IHC) analysis.

Kidney sections of 3-4 *μ*m thickness obtained from paraffin-embedded blocks were routinely stained with Hematoxylin-Eosin, Periodic Acid Schiff (PAS), and Periodic Acid-Silver Methenamine (PASM).

For TLRs immunodetection, paraffin-embedded renal sections were deparaffinized in xylene, dehydrated through a graded ethanol series, and washed in distilled water. The slides were pretreated using heat-induced antigen retrieval performer by immersing the slides in citrate buffer (10 mM, pH 6.0), followed by a period of 25 min of heating to 90°C in a microwave at 600 W with enough buffer to prevent evaporation and drying of the slides during heating and cooling down at room temperature (+22°C) for 20 min. All sections were washed in 0.01 M phosphate buffered saline (PBS) (NaCl 0.138 M; KCl 0.0027 M), pH 7.4, for 3 × 5 min. Endogenous peroxidase activity was blocked by immersing the sections in 0.3% H_2_O_2_ in methanol for 25 min at +22°C; then slides were washed in PBS for 3 × 5 min. Nonspecific binding sites were blocked with 10% normal goat serum (Vector Laboratories Inc., Burlingame, California, USA) diluted in 0.1% bovine serum albumin (BSA) (Sigma-Aldrich, St. Louis, Missouri, USA) during 1-hour incubation at +22°C. Sections were then washed again with PBS.

The primary antibodies used were

- anti-TLR3 (H-125) sc10740 Rabbit Polyclonal Ab. (Santa Cruz Biotechnology Inc., Dallas, Texas, USA) at a 1 : 250 dilution with 0.5% BSA;
- anti-TLR7 (N-20) sc13207 Goat Polyclonal Ab. (Santa Cruz Biotechnology Inc., Dallas, Texas, USA) at a 1 : 120 dilution with 0.5% BSA;
- anti-TLR8 (H-114) sc25467 Rabbit Polyclonal Ab. (Santa Cruz Biotechnology Inc., Dallas, Texas, USA) at a 1 : 150 dilution with 0.5% BSA;
- anti-TLR9 (H-100) sc25468 Rabbit Polyclonal Ab. (Santa Cruz Biotechnology Inc., Dallas, Texas, USA) at a 1 : 400 dilution with 0.5% BSA.

All sections were incubated overnight in a humid box at +4°C. Next day, the sections were washed for 3 × 5 min in PBS.

For each antibody staining, a different slide was used. Slides stained with anti-TLR7 were incubated with secondary UltraTek Polyclonal Anti-Polyvalent Biotinylated Antibody with anti-mouse, anti-rat, anti-rabbit, and anti-Guinea pig specificity, preadsorbed against human (ScyTec Laboratories, Logan, Utah, USA) for 10 minutes at room temperature. Slides stained with anti-TLR3, anti-TLR8, and anti-TLR9 antibodies were incubated with secondary Polyclonal Goat Anti-Rabbit Immunoglobulins/Biotinylated (DAKO, Glostrup, Denmark) at 1 : 400 for 30 at room temperature. All slides were washed for 3 × 5 min in PBS, rinsed, and incubated with streptavidin-peroxidase for 10 min at room temperature.

A peroxidase substrate solution (diaminobenzidine, DAB, 300 *μ*L of 3% H_2_O_2_ and 0.023% 3,3′-diaminobenzidine tetrahydrochloride solution, 35 mg of DAB in 150 mL PBS, ScyTeK Laboratories, Inc., Logan, Utah, USA) was used for 10 min incubation to visualize positive reaction.

Slides were washed in distilled water, dehydrated in graded ethanol series, cleared in xylene, and mounted.

Digital images of IHC-stained kidney samples slides were obtained at 40x magnification (0.0625 *μ*m^2^ per raw image pixel) using a whole slide scanner (ScanScope CS, Aperio) (Aperio Technologies Inc., 1360 Park Center Drive, Vista, California, USA) fitted with a 40x/0.75 Plan Apo. objective lens (Olympus, Center Valley, Pennsylvania, USA). The background illumination levels were calibrated using a prescan procedure. Images were saved in SVS format (Aperio) and retrieved with a file management interface (Spectrum, Aperio).

Most representative glomerular and tubulointerstitial areas of the immunohistochemical reaction on each slide were defined. Three glomerular and three tubulointerstitial areas were selected for analysis in each slide by using the Spectrum Analysis algorithm package and Aperio's IHC Deconvolution Algorithm version 9 (Aperio Technologies, Inc.). Every slide was stained with a single immunohistochemistry reaction and then digitalized and evaluated with the ImageScope algorithm. By using this software, each stain was individually calibrated by analyzing single-stained sections and recording the average red, green, and blue (RGB) optical density (OD) vectors.

The IHC Image Analysis algorithm detects the immunohistochemistry staining for a target chromogen for the individual cells in the selected regions and quantifies their intensity.

**Figure 1:**
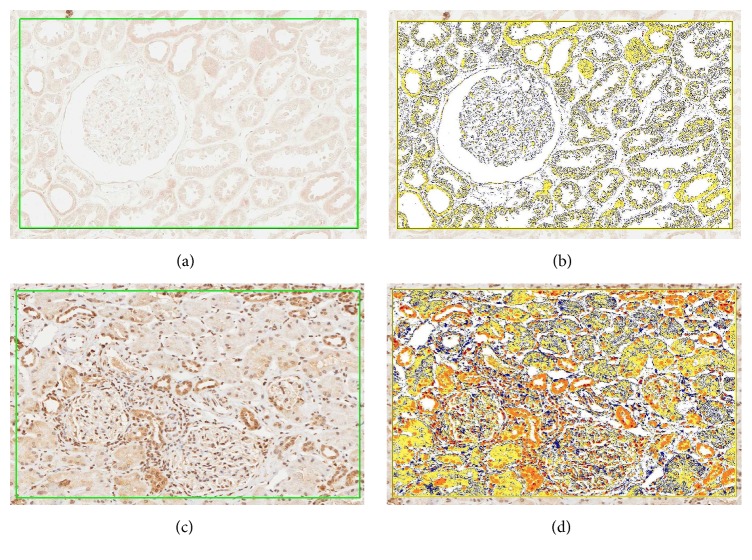
Digital images of immunohistochemistry of kidney biopsy representing the application of Spectrum Analysis algorithm package and Aperio's IHC Deconvolution Algorithm software for the quantification of cells expressing TLRs. Immunohistochemistry for TLR7. Glomerular and tubular areas (20x magnification) of kidney sections from a control (a) and a class IV lupus nephritis patient (c) were selected for the analysis with Spectrum Analysis algorithm package and Aperio's IHC Deconvolution Algorithm (identified by green lines). There is focal background tubular staining. Images (b) and (d) show the same sections after the application of Aperio's IHC Algorithm. Pseudocolors represent staining intensity: blue, no staining; yellow, low intensity/background; orange, medium intensity; and red, high intensity.

Color Deconvolution software individually analyzed DAB staining (deconvolved by its RGB color components) and measured staining intensity only within selected areas. Pseudocolors represent staining intensity: blue, no staining; yellow, low intensities; orange, medium intensities; and red, high intensities in selected areas considered; only high and medium intensity were considered when calculating number of positive cells/mm^2^ expressing each TLR at glomerular level and at tubulointerstitial level or at both (Figure 1). All data were exported in.xls files for statistical analysis.

### 2.2. Statistical Analysis

Data were expressed as mean standard deviation or median and interquartile range (IQR) when they were nonparametric. Mann-Whitney test was used to investigate any difference among groups. Spearman's correlation test was used to assess any possible correlation between TLR expression and clinical parameters. A *p* value of <0.05 was considered significant. IBM SPSS 13 was used for the statistical analysis.

## 3. Results

We enrolled 26 SLE patients with renal involvement.  Table 1 shows demographic and clinical features of the population. Overall, in the 26 SLE patients, we detected a diffuse expression of TLR3 and TLR9 with no significant difference between glomerular staining and tubulointerstitial staining and more pronounced glomerular compared to tubulointerstitial TLR7 and TLR8 expressions (*p* = 0.004 and *p* = 0.03, resp.).

Compared to control, LN sections showed a significantly higher amount of both glomerular and tubulointerstitial TLR9 (*p* = 0.003 and *p* = 0.007) and a higher expression of TLR3 (whole expression, *p* = 0.026, and tubulointerstitial expression, *p* = 0.031) and TLR7 restricted to the tubulointerstitium (*p* = 0.022) (Table 2).

Table 3 shows the number of positive cells/mm^2^ expressing each TLR at glomerular level and at tubulointerstitial level or at both in different LN classes. When comparing TLRs expression among different histological classes, we detected a significantly higher glomerular expression of TLR3 in class III versus class II (*p* = 0.03) and class IV (*p* = 0.03) and higher tubulointerstitial and glomerular TLR9 in class IV versus classes II and III (*p* = 0.02 and *p* = 0.04, and *p* = 0.05 and *p* = 0.01, resp.). We did not find any differences in TLR8 expression among the histological classes.

We found a positive correlation between glomerular TLR9 and activity index (*r* = 0.6; *p* = 0.0063) and between tubular TLR7 and chronicity index (*r* = 0.6; *p* = 0.026); moreover, we detected a positive correlation between tubular TLR9 and R-SLEDAI score (*r* = 0.54; *p* = 0.01) (Table 4).

## 4. Discussion

The results of the present study provide, for the first time, a quantification of glomerular and tubulointerstitial TLRs expressions in kidney sections of patients with LN, confirming their diffuse renal overexpression.

In the last decade, the role of innate immunity in the pathogenesis of LN gained great attention. TLRs are expressed both on leukocytes and on resident renal cells and contribute to the onset of glomerulonephritis and progression of kidney damage by bridging innate and adaptive immune responses [9, 15].

Defective apoptosis and clearance of apoptotic bodies, which are common in SLE patients, determine the release of nucleic acids with subsequent production of ICs; the same nuclear antigens act as ligand for endosomal TLRs (TLR3, TLR7, TLR8, and TLR9) expressed by B cells and antigen presenting cells further contributing to (auto)antibodies production [16].

Several studies on murine lupus support the role of TLRs in glomerulonephritis (reviewed in [4]). Data on renal expression of TLRs in humans are still scant. To date, only few studies analyzed TLRs in kidney sections from LN patients, for the most part focusing on TLR9. High amount of TLR9 was detected in peripheral blood cells of SLE patients, especially in those with active disease [17–21], and TLR9 polymorphism seems to be associated with SLE pathogenesis [22, 23]. Exposure to TLR9 agonist CpG-DNA (and not to TLR7 agonists) induced anti-dsDNA IgG and ICs deposition and was associated with the onset of glomerulonephritis in lupus-prone mice [24]. Moreover, expression of TLR9, protein and mRNA, was observed in mice with glomerulonephritis correlating with proteinuria and interstitial inflammatory infiltrate [24]. The pattern of TLR9 expression in lupus kidney is controversial, since it was demonstrated exclusively at tubular level or both in tubulointerstitium and in glomeruli [5–9]. In 2007, Benigni et al. described the presence of an intense and diffuse TLR9 staining in proximal tubules of class IV LN patients compared with controls, without any glomerular expression; moreover, both sera and purified ICs from SLE patients were able to increase significantly TLR9 mRNA expression in cultured tubular cells [5]. Our findings agree with previously published data demonstrating a wider distribution of TLR9 within the kidney [6–8]: we detected high amount of TLR9, mostly but not exclusively tubulointerstitial, in proliferative histological classes. Similarly, Papadimitraki et al. detected TLR9 in tubulointerstitium of 12 patients with LN and in normal kidneys but they observed also glomerular expression only in the first group [7].

In a murine model of MRL^lpr/lpr^ lupus glomerulonephritis, Patole et al. described the pattern of TLRs expression in mesangial cells and macrophages [25]. The authors demonstrated an increase of TLRs 1–9 mRNAs paralleling the progression of glomerulonephritis: at 5 weeks, only mRNAs for TLRs 1–4 and TLR6 were expressed in the absence of any infiltrating cells; at week 20, there was a wider and stronger expression of TLRs 1–9 [25]. The authors confirmed that mesangial cells were the source of TLRs 1–4 and TLR6 by studying expression and regulation of these receptors in cell culture; cultured macrophages displayed a different expression pattern including all the 9 receptors [25]. Another study demonstrated the expression of mRNA for TLR2, TLR3, TLR4, and TLR6 but not for TLR9 in murine tubular endothelial cell [26].

Ciferska et al. firstly investigated renal expressions of TLRs other than 9, that is, TLR3, TLR7, and TLR8, in humans [6]. In kidney sections obtained from 9 patients with LN, 10 subjects with chronic rejection, and 2 healthy controls, the authors found TLR9 as the only receptor discriminating LN from healthy individuals and controls who did not express TLR9 at all. Kidneys from all the 3 groups diffusely expressed TLR3, TLR7, and TLR8, with LN patients showing more diffuse and stronger staining [6].

Similarly to Ciferska et al., we found a higher expression of whole kidney TLR3 and TLR7 in LN compared to controls; however, we detected TLR9 also in control biopsies, even though in a significantly lower amount; no differences were detectable in TLR8 expression. In addition, among the different histological classes, we found a higher expression of glomerular TLR3 in class III and tubulointerstitial TLR7 in class IV.

The extent of tubulointerstitial involvement seems to predict the renal outcome better than glomerular inflammation severity [27–29]. In the last few years, different authors described the presence of B cells in the kidney infiltrates of patients with LN: in the tubulointerstitium but not in the glomerular tuft [30–32]; the leukocytes initially form a diffuse infiltrate and progressively they may form more complex structures resembling secondary lymphoid organs, anatomically and functionally organized in aggregates of T and B cells or germinal center-like structures (GC) [30, 31, 33]. In MRL^−lpr/lpr^ mice, Herlands et al. demonstrated T cells independent activation of extrafollicular autoreactive B cells requiring TLR7 and TLR9, suggesting that B cell activation could be an initial step of self-tolerance breakdown [34]. More recently, TLR7 signaling was confirmed to be necessary for optimal B cell survival and GC formation both in nonautoimmune and in lupus-prone mice [35]. Interestingly, we detected an overexpression of TLR7 in tubulointerstitial compartment of class IV biopsies. A recent work further pointed out the prognostic role of tubulointerstitial involvement by demonstrating an association of B cells infiltrate within the kidneys and class IV glomerulonephritis, higher activity and chronicity indices, and increased serum creatinine levels [31]. In the light of a possible contribution of different TLRs in different stage of the disease, we found that glomerular TLR9 correlated with activity index, while tubular TLR7 correlated with chronicity index.

To the best of our knowledge, this is the study evaluating the largest cohort of patients described so far. The uniqueness of this study has been the use of a dedicate software to locate and, for the first time, to quantify the number of TLRs-expressing cells within kidney tissue. Moreover, for the first time, we detected a positive correlation between TLR9 and both activity index and Renal-SLEDAI and between TLR7 and chronicity index.

The results of the present study allow only speculating whether the higher amount of TLR expressed in LN at the time of kidney biopsy is related to a different pattern of expression characterizing lupus patients or it results from upregulation of these receptors as a response to kidney injury. This point could be elucidated by further studies evaluating both protein levels and gene expression of TLRs. Indeed, the detection of TLRs in resident renal cells may suggest a role for these receptors as first-line intervention against urinary tract infections; on the other hand, these innate immune modulators might also contribute the onset or worsen an underlying autoimmune disease in predisposed individuals.

In the light of the promising data demonstrating the efficacy of a dual TLR7/TLR9 antagonist and suggesting an “anti-TLR7/9” effect of antimalarials [36], the results of this study further support the role of TLRs as potential target of lupus nephritis. The pattern of TLRs expressions within the kidney observed in the present study seems to suggest a different contribution of the specific receptors to the pathogenesis of LN. However, since we located and quantified TLRs only by immunohistochemistry, we cannot draw any conclusion on the timing of TLRs engagement and their possible role as biomarkers of disease activity and severity. Functional studies are needed to clarify which could be the triggers and timing of the expression of the different TLRs within the kidney.

## 5. Conclusions

In conclusion, the results of this study confirm a wide overexpression of TLR3, TLR7, and TLR9 in kidney sections of LN patients and demonstrate a correlation with clinical and histological indices, further supporting a putative role for these mediators of innate immune response in the pathogenesis of lupus nephritis and suggesting them as potential therapeutic target.

## Figures and Tables

**Table 1:** Demographic and clinical data of the lupus nephritis cohort.

	Patients (*N* = 26)
Gender	
(M : F)	5 : 21
Disease duration (years)	
Mean ± SD	9.97 ± 11.77
Age at biopsy (years)	
Mean ± SD	36.69 ± 12.28
C3 (mg/dl)	
Mean ± SD	60.38 ± 33.47
C4 (mg/dl)	
Mean ± SD	10.59 ± 6.074
Creatinine (mg/dl)	
Median (IQR)	1 (0.7)
Proteinuria g/24 h	
Median (IQR)	736 (1193)
ESR (mm/h)	
Mean ± SD	36.24 ± 26.91
Renal-SLEDAI	
Mean ± SD	6.61 ± 3.38

IQR, interquartile range; ESR, erythrocyte sedimentation rate; SLEDAI, Systemic Lupus Erythematosus Disease Activity Index.

**Table 2:** TLR3, TLR7, TLR8, and TLR9 expressions in kidney section of lupus nephritis patients and healthy controls.

		Lupus nephritis (*n* = 26)	Controls (*n* = 4)	*p*
		Median (IQR)	Median (IQR)	
TLR3	W	1071 (1170)	360 (309)	0.026
	G	667 (476)	234 (211)	ns
	TI	485 (524)	127 (903)	0.031

TLR7	W	1718 (1962)	903 (1278)	ns
	G	983 (1409)	231 (741)	ns
	TI	932 (677)	314 (480)	0.022

TLR8	W	321 (875)	623 (888)	ns
	G	189 (602)	358 (516)	ns
	TI	109 (248)	164 (193)	ns

TLR9	W	667 (1119)	41 (65)	<0.001
	G	372 (646)	14 (41)	0.003
	TI	237 (282)	27 (25)	0.007

W, whole kidney expression; G, glomerular expression; TI, tubulointerstitial expression.

**Table 3:** Number of positive cells/mm^2^ expressing each TLR at glomerular level and at tubulointerstitial level or at both in *class II, class III, and class IV lupus nephritis*.

		Class II	Class III	Class IV	*p*
		*N* = 6	*N* = 9	*N* = 9	
		Median (IQR)	Median (IQR)	Median (IQR)	
TLR3	W	234 (354)	378 (550)	1492 (1414)	ns
	G	272 (183)	187 (661)	480 (881)	Class III versus class II: 0.03 Class III versus class IV: 0.03
	TI	224 (72)	168 (159)	702 (574)	ns

TLR7	W	1617 (332)	3396 (1656)	2531 (1612)	ns
	G	1075 (1174)	1996 (1656)	1414 (892)	ns
	TI	1168 (1192)	1381 (1162)	1127 (778)	ns

TLR8	W	155 (31)	202 (247)	300 (123)	ns
	G	55 (22)	132 (164)	200 (215)	ns
	T	100 (52)	72 (52)	107 (99)	ns

TLR9	W	234 (354)	378 (550)	1492 (1414)	ns
	G	272 (183)	187 (661)	480 (881)	Class IV versus class II: 0.05 Class IV versus class III: 0.01
	TI	224 (72)	168 (159)	702 (574)	Cass IV versus class II: 0.02 Class IV versus class III: 0.04

W, whole kidney expression; G, glomerular expression; TI, tubulointerstitial expression.

**Table 4:** Correlation between kidney TLRs expressions and clinicopathological parameters of lupus nephritis patients.

Toll like receptor	Parameter	*r*	*p*
TLR7	Chronicity index	0.6	0.026
TLR9	Activity index	0.6	0.0063
	R-SLEDAI	0.6	0.01

TLR, Toll Like Receptor; R-SLEDAI, Renal-Systemic Lupus Erythematosus Disease Activity Index.
